# Cardiovascular magnetic resonance based diagnosis of left ventricular non-compaction cardiomyopathy: impact of cine bSSFP strain analysis

**DOI:** 10.1186/s12968-020-0599-3

**Published:** 2020-01-30

**Authors:** John G. Dreisbach, Shobhit Mathur, Christian P. Houbois, Erwin Oechslin, Heather Ross, Kate Hanneman, Bernd J. Wintersperger

**Affiliations:** 1 Department of Medical Imaging, Peter Munk Cardiac Centre, University Health Network, Toronto General Hospital, 585 University Avenue, Toronto, M5G 2N5 Ontario Canada; 2 Department of Medical Imaging, University of Toronto, Toronto, Ontario Canada; 3 Division of Cardiology, Peter Munk Cardiac Centre, University Health Network, Toronto, Ontario Canada; 4 Department of Medicine, University of Toronto, Toronto, Ontario Canada

**Keywords:** Isolated noncompaction of the ventricular myocardium, Cardiovascular magnetic resonance, Myocardial strain, Feature tracking, Ventricular dysfunction

## Abstract

**Background:**

Investigation of the myocardial strain characteristics of the left ventricular non-compaction (LVNC) phenotype with cardiovascular magnetic resonance (CMR) feature tracking.

**Methods:**

CMR cine balanced steady-state free precession data sets of 59 retrospectively identified LVNC phenotype patients (40 years, IQR: 28–50 years; 51% male) and 36 healthy subjects (39 years, IQR: 30–47 years; 44% male) were evaluated for LV volumes, systolic function and mass. Hypertrabeculation in patients and healthy subjects was evaluated against established CMR diagnostic criteria. Global circumferential strain (GCS), global radial strain (GRS) and global longitudinal strain (GLS) were evaluated with feature-tracking software. Subgroup analyses were performed in patients (*n* = 25) and healthy subjects (*n* = 34) with normal LV volumetrics, and with healthy subjects (*n* = 18) meeting at least one LVNC diagnostic criteria.

**Results:**

All LVNC phenotype patients, as well as a significant proportion of healthy subjects, met morphology-based CMR diagnostic criteria: non-compacted (NC): compacted myocardial diameter ratio > 2.3 (100% vs. 19.4%), NC mass > 20% (100% vs. 44.4%) and > 25% (100% vs. 13.9%), and NC mass indexed to body surface area > 15 g/m^2^ (100% vs. 41.7%). LVNC phenotype patients demonstrated reduced GRS (26.4% vs. 37.1%; *p* < 0.001), GCS (− 16.5% vs. -20.5%; *p* < 0.001) and GLS (− 14.6% vs. -17.1%; *p* < 0.001) compared to healthy subjects, with statistically significant differences persisting on subgroup comparisons of LVNC phenotype patients with healthy subjects meeting diagnostic criteria. GCS also demonstrated independent and incremental diagnostic value beyond each of the morphology-based CMR diagnostic criteria.

**Conclusions:**

LVNC phenotype patients demonstrate impaired strain by CMR feature tracking, also present on comparison of subjects with normal LV volumetrics meeting diagnostic criteria. The high proportion of healthy subjects meeting morphology-based CMR diagnostic criteria emphasizes the important potential complementary diagnostic value of strain in differentiating LVNC from physiologic hypertrabeculation.

## Background

Left ventricular non-compaction cardiomyopathy (LVNC) is a rare cardiomyopathy that can occur in isolation or in association with other congenital and acquired cardiac pathologies [1]. It is characterised by two distinct layers of the left ventricular (LV) myocardium – an endocardial layer of heavily hypertrabeculated myocardium with deep inter-trabecular recesses, and an abnormally thin epicardial layer of compacted myocardium [2]. Despite controversies regarding the development of the myocardial phenotype of LVNC, the abnormal morphology characteristically involves the final segments to undergo compaction during embryogenesis – the apical inferior and lateral segments, with variable basal extension [3, 4].

Previously considered a rare anomaly, the widespread availability and advances in image quality of echocardiography and cardiovascular magnetic resonance (CMR) imaging and increased awareness have led to increasingly frequent recognition of the LVNC phenotype [1]. However, variable clinical manifestations and a wide spectrum of natural history have been observed, ranging from an asymptomatic imaging finding to life-threatening conditions including heart failure, arrhythmia and systemic thromboembolism [5].

Multiple validated morphology-based echocardiographic diagnostic criteria have evolved since the early descriptions of LVNC [2, 6–8] with a similar development of diagnostic criteria using CMR [9–11]. However, despite increasingly sophisticated methods of describing the myocardial phenotype and quantifying the degree of non-compaction, significant limitations in the clinical utility of morphology-based diagnostic criteria persist. Importantly, there is a tendency to over-diagnose LVNC among healthy subjects [12], without significant adverse outcomes on long-term follow-up [13]. Indeed, it is critical to distinguish patients with physiologic remodelling of the myocardium from those with pathologic remodelling or LVNC [14].

In order to expand beyond pure morphologic features and global volumetric parameters of cardiac function in the diagnosis and assessment of ischemic and non-ischemic cardiomyopathies, quantitative assessment of myocardial deformation by strain analysis has developed over recent years. Speckle-tracking echocardiography (STE) has demonstrated abnormal patterns of LV myocardial strain and torsion in LVNC including decreased radial, circumferential and longitudinal strain, decreased LV twist and abnormal patterns of ventricular rotation [15–18]. Strain analysis by CMR feature tracking (FT) using routine cine balanced steady-state free precession (bSSFP) data sets can reproducibly quantify myocardial deformation across multiple parameters [19]. However, there is limited data available on the characteristics of myocardial deformation in LVNC assessed by CMR FT. In light of the limitations of current morphology-based diagnostic criteria, this study investigated functional parameters including myocardial strain characteristics among LVNC phenotype patients and healthy subjects with CMR FT to explore potential complementary diagnostic utility. Ancillary aims included investigating whether potential differences in myocardial strain persist independent of LV volumetrics, parameters known to affect strain [20], between LVNC phenotype patients and healthy subjects, including subjects with and without features of ‘physiologic’ hypertrabeculation.

## Methods

### Study population

**Fig. 1:**
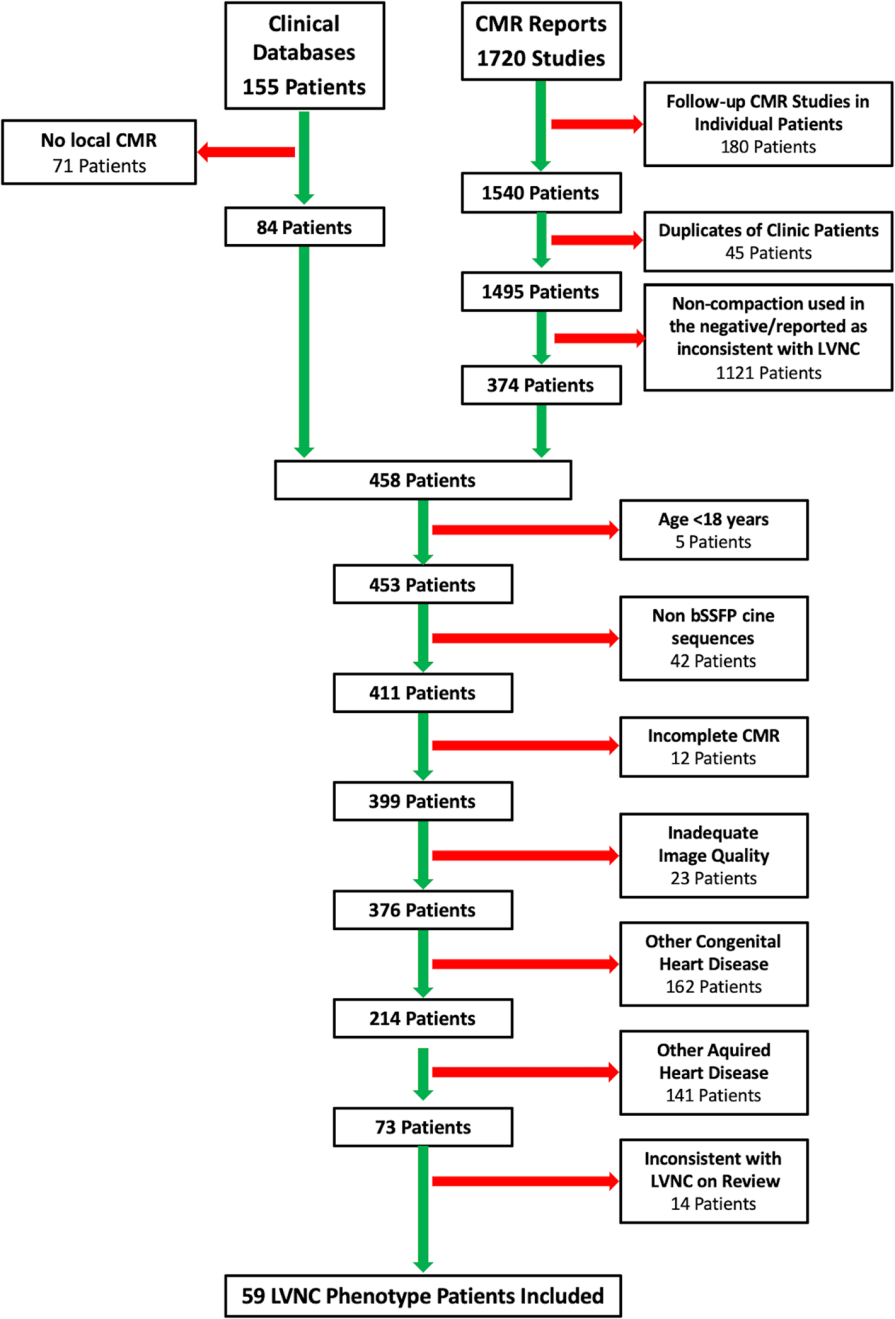
Retrospective Patient Identification. CMR = cardiovascular magnetic resonance; LVNC = left ventricular non-compaction cardiomyopathy; bSSFP = balanced steady-state free precession

This single centre retrospective cohort study identified patients with isolated LVNC phenotype from heart failure and adult congenital cardiac disease clinic databases, as well as a Boolean search of CMR reports between June 2008 and July 2017. The keyword search of CMR reports included the terms ‘non-compaction’ and/or ‘hypertrabeculation’, and returned cases were qualitatively reviewed by two readers (JGD and BJW) to confirm findings consistent with the LVNC phenotype. Patient exclusion criteria included age < 18 years, incomplete cine bSSFP coverage of the LV, CMR studies with limited image quality, and any concurrent congenital or acquired heart disease with the exception of idiopathic dilated cardiomyopathy with hypertrabeculation. Patient identification and study inclusion is summarized in Fig. 1. Healthy volunteers with no known history of cardiovascular disease that were originally recruited as part of the EMBRACE-MRI (clinicaltrials.gov: NCT02306538) and MAFIO (clinicaltrials.gov: NCT02090699) studies were included as a healthy control population [21]. LVNC phenotype patients and healthy subjects with normal LV volumetrics were separated for subgroup analysis and labelled subgroup A and B, respectively. Healthy subjects were further subdivided into those meeting none of the CMR diagnostic criteria for LVNC (subgroup C) and those meeting one or more criteria (subgroup D). The following subgroup comparisons were performed: subgroup A vs. B; A vs. C; A vs. D; and C vs. D. The study protocol conformed to the Declaration of Helsinki and was approved by the institutional research ethics board (University Health Network Research Ethics Board). Written informed consent was obtained from all healthy subjects and was waived for the patient cohort.

### CMR technique

CMR scans were performed on one of four scanners: Magnetom Skyra^fit^/Verio (3 T) or Avanto/Avanto^fit^ (1.5 T) (Siemens Healthineers, Erlangen, Germany). All studies included retrospectively gated LV 2-, 3- and 4-chamber long-axis (LAX) single-slice and short-axis oblique (SAO) base-to-apex stack cine bSSFP sequences with 6–8 mm slice thickness, 2 mm slice gap, pixel size ≤1.8 × 1.8 mm^2^, and a temporal resolution < 50 ms.

### CMR analysis

Analysis of LV volumes, systolic function, mass and strain was performed using commercially post-processing software (cvi^42^, Circle Cardiovascular Imaging, Calgary, Canada) by a single cardiac-fellowship trained radiologist with 2 years of cardiac imaging experience (JGD) blinded to pre-existing clinical and radiological information.

### LV Volumetrics

Abnormality of LV volumetrics (i.e. dilation, impaired systolic function and hypertrophy) was assessed against gender-specific reference ranges validated for cine bSSFP sequences [22]. Definitions for males and females respectively included: LV dilation as an EDVi (end-diastolic volume (EDV) indexed to BSA) > 112 ml/m^2^ and > 99 ml/m^2^; impaired LV systolic function as an ejection fraction (EF) < 55 and < 54%; LV hypertrophy as a MASSi (mass indexed to BSA) > 83 g/m^2^ and > 67 g/m^2^.

### CMR diagnostic criteria for LVNC

The following validated CMR diagnostic criteria for LVNC were assessed in all patients and volunteers: non-compacted (NC): compacted (C) myocardium diameter ratio > 2.3 in end-diastole [9], NC > 20% of LV mass [10], NC > 25% of LV mass, and NC indexed mass > 15 g/m^2^ [11].

Measurements of NC and C thickness for the calculation of the NC:C ratio were performed on end-diastolic SAO and/or LAX cine images with the myocardium orthogonal to the plane of measurement.

A previously validated technique was employed to measure the NC myocardial mass [10, 11]. A semi-automated threshold-based technique was used to contour the end-diastolic endocardial border of the NC myocardium along with standard epicardial contours to measure the ‘global myocardial mass’, without accounting for the intervening blood pool within the inter-trabecular recesses of the NC myocardium. The NC mass was then calculated by subtracting both the C myocardial mass (measured with standard endo- and epicardial contours) and papillary muscle mass from the global myocardial mass.

**Fig. 2:**
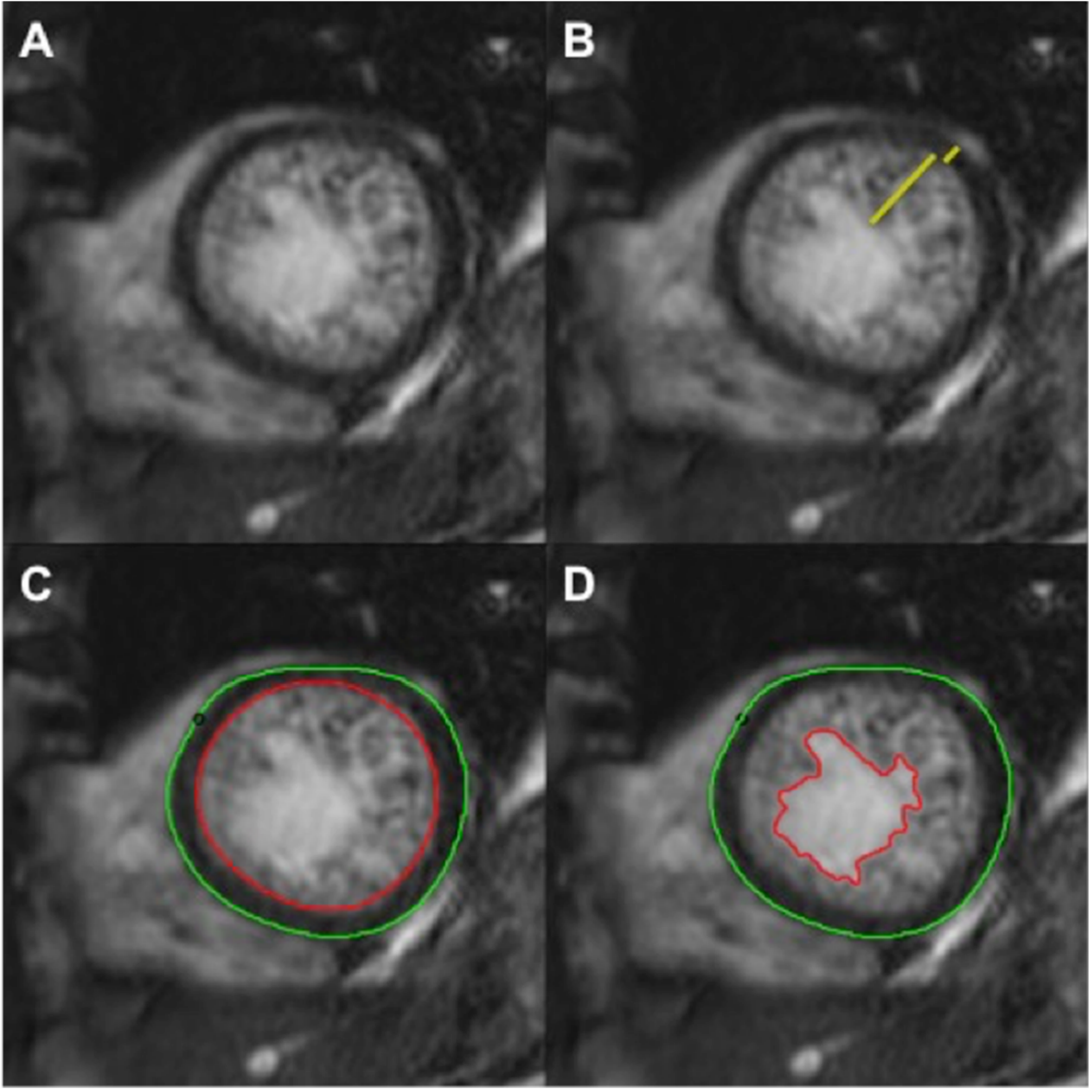
CMR Diagnostic Criteria for LVNC. **a** CMR short-axis cine bSSFP image of the apical LV demonstrating severely thickened myocardium with prominent trabeculations and deep intertrabecular recesses in a 49-year-old female with a history of ventricular tachycardia and LVNC phenotype. **b** Measurement of the maximum non-compacted (NC): compacted myocardium diameter ratio. **c** Standard epicardial contours and endocardial contours at the border of the compacted and NC myocardium for measurement of the compacted myocardial mass. **d** Endocardial contours at the border of the NC myocardium and non-trabeculated cavity for measurement of the global myocardial mass (compacted and non-compacted myocardial mass). CMR = cardiovascular magnetic resonance; LVNC = left ventricular non-compaction cardiomyopathy; bSSFP = balanced steady-state free precession; LV = left ventricle

Methods of assessment of the NC myocardium are illustrated in Fig. 2.

### CMR Strain Analysis

#### Radial, circumferential and longitudinal strain

Radial and circumferential strain were measured across the full thickness of the myocardium from the SAO cine bSSFP stack following application of end-diastolic endo- and epicardial contours with superior and inferior insertion point markers to three selected slices at representative basal, mid-ventricular and apical levels. The selected basal slices included circumferentially complete myocardium throughout the cardiac cycle, the selected apical slices avoided obliquely oriented myocardium, and the selected mid-ventricular slices were equidistant between the selected basal and apical slices at the level of the papillary muscles. Global radial strain (GRS) and global circumferential strain (GCS) were calculated from the average of the peak strain of the three selected SAO slices.

Longitudinal strain was measured across the full thickness of the myocardium from the LV 2-, 3- and 4-chamber cine bSSFP single-slice images by applying end-diastolic endo- and epicardial contours with a T-bar defining the mitral valve plane and LV apex. Global longitudinal strain (GLS) was calculated from the average of the peak strain of the three LAX slices.

#### Twist and torsion

Twist was measured as the rotational circumferential displacement (measured in degrees) at the selected basal and apical SAO slices. Torsion was calculated as the difference between basal and apical rotation (i.e. twist) divided by the distance between the two slices along the long axis of the LV.

### Statistical analysis

All statistical analysis was performed using STATA v14.1 (StataCorp, College Station, Texas) and statistical significance was defined as a two-tailed *p*-value of < 0.05. Categorical variables are described as number and percentage and continuous variables are presented as median and interquartile range (IQR). Testing for normal distribution was performed using the Shapiro-Wilk test. Comparison of categorical variables was performed using Fisher’s exact test, normally distributed continuous variables with the independent samples t-test, and non-normally distributed continuous variables with the Wilcoxon rank sum test. Global strain values were evaluated with receiver operating characteristic (ROC) analyses.

To assess whether strain parameters are independent of EF in the subgroup A vs. D comparison, individual bivariable logistic regression models were fitted with respective strain measures and EF, and to assess for incremental diagnostic value, the likelihood ratio test was used to compare nested models with EF alone versus a model with GCS added. To assess the independence and incremental diagnostic value of GCS against each of the morphologic CMR diagnostic criteria for LVNC in differentiating all healthy volunteers and LVNC phenotype patients, individual bivariable logistic regression models were fitted with GCS and each diagnostic criteria, and the likelihood ratio test was used to compare nested models with each diagnostic criteria alone versus models with GCS added.

## Results

### Study population

One hundred fifty-five study candidates were identified from the clinical databases and 1720 exams from the CMR report database. A total of 59 LVNC phenotype patients were included following elimination of duplicate patients and CMR reports using keywords in the negative (e.g. “no evidence of non-compaction”), application of exclusion criteria, and review of CMR data sets studies by the two readers. All 59 (100%) of the patients met each of the four CMR diagnostic criteria for LVNC. LVNC phenotype patients included 30 males (51%) with a median age of 40 years (IQR: 28–50 years). The control group of 36 healthy subjects included 16 males (44%) with a median age of 39 years (IQR: 30–47 years). Demographic characteristics are detailed in Table 1. There were no significant differences in sex (*p* = 0.673), age (*p* = 0.866) or BSA (*p* = 0.863) between all healthy subjects and LVNC phenotype patients. All healthy subjects (*n* = 36/36) were scanned at 1.5 T. Of the LVNC phenotype patients, 61% (*n* = 36/59) were scanned at 1.5 T and 39% (*n* = 23/59) at 3.0 T.

**Table 1:** Demographic Characteristics and LV Volumetrics

	All patients and volunteers			Subgroup A	Subgroup B	Subgroup C	Subgroup D	A vs. B	A vs. C	A vs. D	C vs. D
	All LVNC phenotype patients	All healthy subjects	*p*-values	LVNC phenotype patients (normal LV volumetrics)	Healthy subjects (normal LV volumetrics)	Healthy subjects (normal LV volumetrics, not meeting any LVNC criteria)	Healthy subjects (normal LV volumetrics, meeting one or more LVNC criteria)	*p*-values	*p*-values	*p*-values	*p*-values
	(*n* = 59)	(*n* = 36)		(*n* = 25)	(*n* = 34)	(*n* = 16)	(*n* = 18)				
Male^a^	30 (51%)	16 (44%)	0.673	9 (36%)	15 (44%)	4 (25%)	11 (61%)	0.598	0.513	0.130	0.045
Age (years)^b^	40.3 (28.4, 50.5)	39.0 (30.2, 46.6)	0.866	33.2 (22.7, 42.5)	39.0 (30.4, 47)	44.7 (38.0, 57.9)	34.3 (30.0, 40.8)	0.089	0.017	0.588	0.012
BSA (m^2^)^c^	1.87 (1.64, 2.08)	1.82 (1.63, 2.02)	0.863	1.69 (1.55, 1.87)	1.82 (1.63, 1.99)	1.72 (1.63, 1.89)	1.94 (1.71, 2.05)	0.118	0.527	0.045	0.198
Heart Rate (bpm)^b^	65 (56, 74)	62 (59, 706)	0.466	66 (57, 72)	63 (59, 71)	64 (60, 70)	62 (56, 71)	0.545	0.820	0.445	0.469
EDV (ml)^b^	173 (143, 203)	152 (122, 180)	0.054	143 (127, 155)	145 (122, 176)	137 (120, 155)	171 (122, 197)	0.439	0.669	0.102	0.079
EDVI (ml/m^2^)^b^	93.0 (80.7, 102.9)	84.2 (72.5, 95.6)	0.036	83.0 (74.8, 91.7)	83.8 (71.4, 92.6)	78.4 (69.3, 85.5)	88.3 (81.1, 102.1)	0.902	0.181	0.153	0.032
ESV (ml)^b^	79 (58, 103)	54 (45, 69)	< 0.001	58 (53, 70)	53 (44, 68)	50 (40, 56)	68 (48, 74)	0.390	0.019	0.431	0.027
ESVI (ml/m^2^)^b^	42.4 (33.6, 53.1)	30.2 (25.7, 37.8)	< 0.001	34.1 (31.1, 39.2)	30.1 (25.6, 36.0)	29.8 (22.8, 30.6)	33.9 (25.8, 39.6)	0.051	0.002	0.787	0.019
SV (ml)^b^	86 (76, 107)	95 (77, 118)	0.121	80 (74, 91)	93 (77, 116)	87 (795, 99)	103 (76, 122)	0.051	0.219	0.046	0.178
SVI(ml/m^2^)^c^	49.2 (41.3, 52.7)	54.1 (46.8, 59.0)	0.796	49.5 (43.9, 52.6)	53.3 (46.7, 58.3)	50.1 (46.6, 55.2)	56.5 (47.0, 59.9)	0.033	0.205	0.008	0.125
EF (%)^b^	54 (49, 59)	63 (60, 66)	< 0.001	59 (57, 60)	63 (60, 66)	65 (63, 68)	61 (59, 64)	< 0.001	< 0.001	0.004	0.023
CO (l/min)^b^	5.66 (4.74, 6.96)	5.85 (4.90, 7.10)	0.485	5.13 (4.53, 6.13)	5.85 (4.91, 7.07)	5.60 (4.96, 6.75)	6.30 (4.89, 7.78)	0.163	0.364	0.161	0.512
LVM (g)^b^	105 (82, 136)	98 (84, 128)	0.747	82 (67, 99)	98 (83, 128)	93 (81, 102)	108 (84, 142)	0.018	0.181	0.011	0.121
LVMI (g/m^2^)^b^	54.9 (48.9, 66.2)	55.4 (50.1, 65.5)	0.777	49.5 (43.7, 54.2)	55.0 (50.0, 64.7)	52.0 (48.9, 56.8)	55.8 (50.1, 69.7)	0.012	0.157	0.007	0.113

Data presented as n (%) or median (IQR)

*LV* left ventricle, *LVNC* left ventricular non-compaction cardiomyopathy, *BSA* body surface area, *bpm* beats per minute, *EDV* end-diastolic volume, *EDVI* end-diastolic volume indexed to BSA, *ESV* end-systolic volume, *ESVI* end-systolic volume indexed to BSA, *SV* stroke volume, *SVi* stroke volume indexed to BSA, *EF* ejection fraction, *CO* cardiac output, *LVM* left ventricular mass, *LVMI* LVM indexed to BSA

^a^Fisher’s exact test

^b^Wilcoxon rank sum test

^c^Independent samples t-test

### Subgroup analyses

Subgroups of subjects with normal LV volumetrics included 25 LVNC phenotype patients (subgroup A) and 34 healthy subjects (subgroup B); of the two healthy subjects excluded, both had borderline LV dilation and one had borderline impaired EF. Among the healthy subjects 16 met none of the CMR diagnostic criteria for LVNC (subgroup C) and 18 met one or more criteria (subgroup D).

#### Subgroup a vs. B

Comparison of LVNC phenotype patients (*n* = 25) and healthy suybjects (*n* = 34) with normal LV volumetrics revealed no statistically significant differences in sex (*p* = 0.598), age (*p* = 0.089), or BSA (*p* = 0.118).

#### Subgroup a vs. C

Comparison of LVNC phenotype patients with normal LV volumetrics (*n* = 25) and healthy subjects with normal LV volumetrics not meeting any LVNC criteria (*n* = 16) revealed a significant difference in age (*p* = 0.017) but no statistically significant differences in sex (*p* = 0.513), or BSA (*p* = 0.527).

#### Subgroup a vs. D

Comparison of LVNC phenotype patients with normal LV volumetrics (*n* = 25) and healthy subjects with normal LV volumetrics meeting one or more LVNC criteria (*n* = 18) revealed a significant difference in BSA (*p* = 0.045) but no statistically significant differences in sex (*p* = 0.130) or age (*p* = 0.588).

#### Subgroup C vs. D

Comparison between healthy subjects with normal LV volumetrics not meeting any of the LVNC criteria (*n* = 16) and those meeting one or more criteria (*n* = 18) revealed significant differences in sex (*p* = 0.045) and age (*p* = 0.012), but no statistically significant difference in BSA (*p* = 0.198).

### LV Volumetrics

Results for LV volumetrics are detailed in Table 1. Statistically significant differences were observed between all LVNC phenotype patients and healthy subjects for EDVi (*p* = 0.036), ESV (*p* < 0.001), ESVi (end-systolic volume indexed to BSA) (*p* < 0.001) and EF (*p* < 0.001). The remaining LV volumetric parameters showed no statistically significant difference.

#### Subgroup a vs. B

Statistically significant differences were demonstrated in EF (*p* < 0.001), mass (*p* = 0.018), MASSi (*p* = 0.012) and SVi (stroke volume indexed to BSA) (*p* = 0.033).

#### Subgroup a vs. C

Statistically significant differences were demonstrated in EF (*p* < 0.001), ESV (*p* = 0.019) and ESVi (*p* = 0.002).

#### Subgroup a vs. D

Statistically significant differences were demonstrated in EF (*p* < 0.004), SV (*p* = 0.046), SVi (*p* = 0.008), mass (*p* = 0.011) and MASSi (*p* = 0.007).

#### Subgroup C vs. D

Statistically significant differences were demonstrated in EF (*p* < 0.023), EDVi (*p* = 0.032), ESV (*p* = 0.027) and ESVi (*p* = 0.019).

### CMR diagnostic criteria for LVNC

Results for CMR diagnostic criteria for LVNC are detailed in Table 2. Although statistically significant differences were observed between healthy subjects and LVNC phenotype patients across all diagnostic criteria and measures of NC mass, a sizeable minority of the control group also met diagnostic criteria for LVNC. Seven healthy subjects (19.4%) demonstrated at least one segment of hypertrabeculation with an NC:C diameter ratio > 2.3, with 16 (44.4%) exceeding an NC mass of 20% (median 19.5%; IQR 17.2, 23.3%) and 5 (13.9%) exceeding 25% of the global LV myocardium, as well as 15 (41.7%) with an indexed NC mass > 15 g/m^2^ (median 13.6 g/m^2^; IQR 10.5, 17.2).

**Table 2:** CMR Diagnostic Criteria for LVNC

	All patients and healthy subjects			Subgroup A	Subgroup B	Subgroup C	Subgroup D	A vs. B	A vs. C	A vs. D	C vs. D
	All LVNC phenotype patients	All healthy subjects	*p*-values	LVNC phenotype patients (normal LV volumetrics)	Healthy subjects (normal LV volumetrics)	Healthy subjects (normal LV volumetrics, not meeting any LVNC criteria)	Healthy subjects (normal LV volumetrics, meeting one or more LVNC criteria)	*p*-values	*p*-values	*p*-values	*p*-values
	(*n* = 59)	(*n* = 36)		(*n* = 25)	(*n* = 34)	(*n* = 16)	(*n* = 18)				
Number of NC segments^b^	8 (6, 9)	0 (0, 0)	< 0.001	8 (6, 9)	0 (0, 0)	0 (0, 0)	0 (0, 1)	< 0.001	< 0.001	< 0.001	0.013
Maximum NC:C ratio^b^	3.8 (3.0, 4.3)	1.2 (0.9, 1.6)	< 0.001	3.5 (3.2, 4.3)	1.1 (0.9, 1.3)	1.0 (0.8, 1.2)	1.2 (0.9, 2.5)	< 0.001	< 0.001	< 0.001	0.182
NC:C > 2.3^a^	59 (100%)	7 (19.4%)	< 0.001	25 (100%)	6 (17.7%)	0 (0%)	6 (33.3%)	< 0.001	< 0.001	< 0.001	0.020
Percentage of NC mass (%)^b^	40.8 (35.7, 45.9)	19.5 (17.2, 23.3)	< 0.001	42.2 (38.7, 47.1)	19.3 (17.1, 23.6)	17.2 (14.8, 18.5)	23.3 (20.1, 30.7)	< 0.001	< 0.001	< 0.001	< 0.001
NC mass > 20%^a^	59 (100%)	16 (44.4%)	< 0.001	25 (100%)	14 (41.2%)	0 (0%)	14 (77.8%)	< 0.001	< 0.001	0.025	< 0.001
NC mass > 25%^a^	59 (100%)	5 (13.9%)	< 0.001	25 (100%)	5 (14.7%)	0 (0%)	5 (27.8%)	< 0.001	< 0.001	< 0.001	0.046
NC mass/BSA (g/m^2^)^b^	36.1 (32.3, 42.9)	13.6 (10.5, 17.2)	< 0.001	35.1 (30.9, 39.2)	12.7 (10.4, 17.0)	10.0 (8.8, 11.6)	17.0 (15.0, 23.7)	< 0.001	< 0.001	< 0.001	< 0.001
NC mass > 15 g/m^2a^	59 (100%)	15 (41.7%)	< 0.001	25 (100%)	13 (38.2%)	0 (0%)	13 (72.2%)	< 0.001	< 0.001	0.009	< 0.001

Data presented as n (%) or median (IQR)

NC:C >2.3 = maximum end-diastolic non-compacted (NC): compacted (C) myocardium diameter ratio >2.3 in at least one segment.

*CMR* cardiovascular magnetic resonance, *LVNC* left ventricular non-compaction cardiomyopathy, *LV* left ventricle, *NC* non-compacted myocardium, *C* compacted myocardium, *BSA* body surface area

^a^Fisher’s exact test

^b^Wilcoxon rank sum test

#### Subgroup analyses

For subgroup comparisons A vs. B, A vs. C and A vs. D, statistically significant differences persisted across all LVNC diagnostic criteria and measures of NC myocardium. On comparison of healthy subjects with normal LV volumetrics meeting one or more LVNC criteria and those meeting none (subgroup C vs. D), only the maximum NC:C ratio did not show statistically significant difference. Of the diagnostic criteria for LVNC met by 18 (53%) healthy subjects with normal LV volumetrics, 5 healthy subjects (28%) met four criteria, 1 (6%) met three criteria, 6 (33%) met two criteria, and 8 (44%) met one of the four criteria.

### CMR strain analysis

Results for strain analysis and torsion are detailed in Table 3.

**Table 3:** Strain and Torsion

	All patients and healthy subjects			Subgroup A	Subgroup B	Subgroup C	Subgroup D	A vs. B	A vs. C	A vs. D	C vs. D
	All LVNC phenotype patients	All healthy volunteers	*p*-values	LVNC phenotype patients (normal LV volumetrics)	Healthy subjects (normal LV volumetrics)	Healthy subjects (normal LV volumetrics, not meeting any LVNC criteria)	Healthy subjects (normal LV volumetrics, meeting one or more LVNC criteria)	*p*-values	*p*-values	*p*-values	*p*-values
	(*n* = 59)	(*n* = 36)		(*n* = 25)	(*n* = 34)	(*n* = 16)	(*n* = 18)				
GRS (%)^a^	26.4 (21.8, 32.7)	37.1 (33.4, 43.1)	< 0.001	31.2 (28.2, 34.7)	37.5 (34.2, 43.5)	40.2 (35.5, 44.3)	35.8 (33.1, 40.8)	< 0.001	< 0.001	0.006	0.103
Basal RS (%)^a^	26.8 (21.2, 30.7)	34.6 (31.0, 39.1)	< 0.001	28.8 (27.4, 36.1)	34.6 (31.4, 39.1)	35.3 (33.9, 40.9)	32.0 (30.3, 35.6)	0.004	0.002	0.112	0.044
Mid RS (%)^a^	23.9 (19.7, 28.9)	29.9 (24.9, 35.9)	< 0.001	28.5 (24.7, 31.8)	30.5 (25.8, 36.2)	32.4 (29.5, 37.1)	26.6 (24.0, 35.5)	0.065	0.008	0.516	0.087
Apical RS (%)^a^	29.4 (24.0, 37.3)	46.4 (40.1, 56.4)	< 0.001	33.3 (29.4, 42.2)	47.3 (41.8, 58.2)	49.2 (42.8, 59.8)	45.6 (40.3, 51.4)	< 0.001	< 0.001	0.002	0.451
GCS (%)^b^	−16.5 (−14.7, −18.6)	−20.5 (−19.1, −22.1)	< 0.001	−18.5 (−16.8, −19.8)	−20.6 (−19.3, −22.1)	−21.5 (−20.1, −22.9)	−20.0 (−18.8, −21.7)	< 0.001	< 0.001	0.005	0.073
Basal CS (%)^b^	−16.3 (−14.2, −18.1)	−19.6 (−18.4, −21.1)	< 0.001	−17.4 (−16.6, −20.0)	−19.6 (−18.4, −21.2)	−20.1 (−19.3, −21.7)	−18.5 (− 18.2, − 20.0)	0.003	0.002	0.072	0.027
Mid CS (%)^b^	−15.5 (−13.4, −17.8)	− 18.3 (− 16.1, − 20.3)	< 0.001	−17.5 (− 16.1, − 18.9)	− 18.6 (− 16.5, − 20.4)	− 19.4 (− 18.1, − 21.0)	−17.2 (−15.9, − 19.9)	0.061	0.005	0.658	0.079
Apical CS (%)^b^	−17.8 (−15.4, − 19.9)	−23.4 (− 21.9, −25.9)	< 0.001	− 19.4 (− 17.8, − 22.2)	−23.7 (− 22.3, − 26.3)	−24.4 (− 22.6, −26.6)	−23.0 (− 22.0, −24.5)	< 0.001	< 0.001	< 0.001	0.270
GLS (%)^b^	−14.6 (−12.9, − 16.5)	− 17.1 (− 16.1, − 18.7)	< 0.001	− 16.4 (− 15.3, − 17.3)	−17.1 (− 16.2, − 19.0)	−17.2 (− 16.7, − 19.6)	− 16.9 (− 16.1, − 18.3)	0.011	0.009	0.099	0.143
4-chamber LS (%)^b^	−14.7 (− 12.9, − 16.9)	−18.1 (− 16.8, − 19.5)	< 0.001	−16.0 (− 14.6, − 16.6)	−16.7 (− 15.4, − 18.4)	− 17.6 (− 16.2, − 20.4)	−16.2 (− 15.0, − 17.3)	0.021	0.003	0.313	0.032
3-chamber LS (%)^b^	−14.7 (− 12.5, − 16.5)	−17.5 (− 15.9, − 19.1)	< 0.001	− 16.4 (− 14.9, − 17.9)	− 17.6 (− 16.1, − 19.1)	−17.8 (− 16.4, − 19.3)	− 17.3 (− 16.0, − 18.6)	0.046	0.048	0.168	0.427
2-chamber LS (%)^b^	−14.8 (−13.2, − 16.7)	− 17.6 (− 16.3, − 19.3)	< 0.001	− 16.7 (− 14.8, − 18.5)	−17.6 (− 16.4, − 19.2)	−17.7 (− 16.3, − 18.7)	−17.5 (− 16.4, − 19.4)	0.034	0.109	0.055	0.717
Global Torsion (°/cm)^b^	1.01 (0.68, 1.43)	0.94 (0.74, 1.54)	0.765	1.23 (0.99, 1.47)	0.98 (0.78–1.55)	1.01 (0.86, 1.75)	0.93 (0.71, 1.42)	0.470	0.852	0.184	0.178

Data presented as median (IQR)

*LVNC* left ventricular non-compaction cardiomyopathy, *LV* left ventricle, *GRS* global radial strain, *RS* radial strain, *GCS* global circumferential strain, *CS* circumferential strain, *GLS* global longitudinal strain, *LS* longitudinal strain

^a^Independent samples t-test

^b^Wilcoxon rank sum test

#### Radial, circumferential and longitudinal strain

**Fig. 3:**
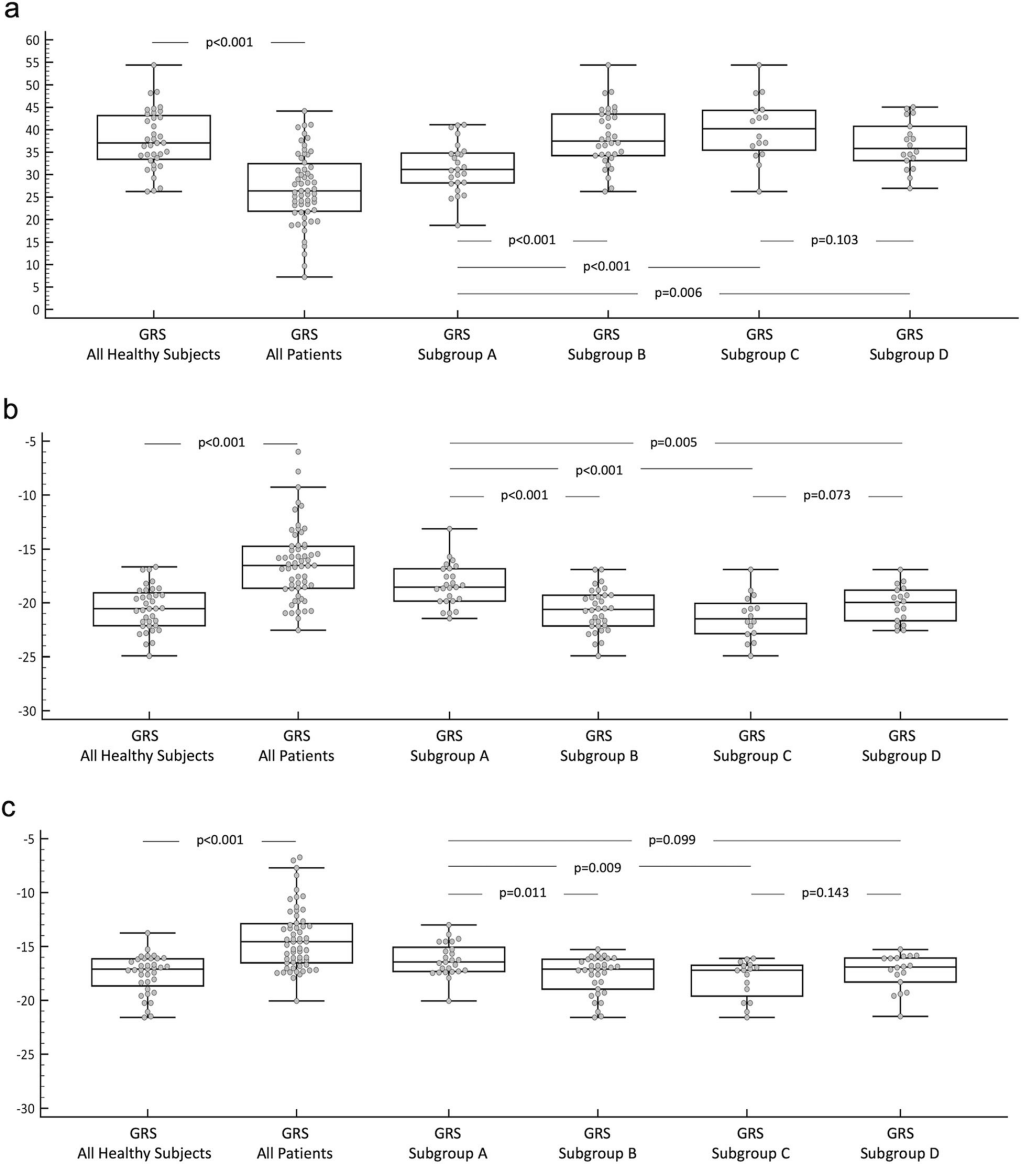
Strain: Box Plots. Box plots comparing **a** GRS, **b** GCS and **c** GLS between: all LVNC phenotype patients and healthy subjects; Subgroup A vs. B (LVNC phenotype patients with normal LV volumetrics and healthy subjects with normal LV volumetrics); Subgroup A vs. C (LVNC phenotype patients with normal LV volumetrics and healthy subjects with normal LV volumetrics not meeting any CMR diagnostic criteria for LVNC); Subgroup A vs. D (LVNC phenotype patients with normal LV volumetrics and healthy subjects with normal LV volumetrics meeting at least one of the CMR diagnostic criteria for LVNC); Subgroup C vs. D (healthy subjects with normal LV volumetrics meeting none of the CMR diagnostic criteria for LVNC and those meeting at least one criteria). GRS = global radial strain; GCS = global circumferential strain; GLS = global longitudinal strain; LVNC = left ventricular non-compaction cardiomyopathy; LV = left ventricle; CMR = cardiovascular magnetic resonance

**Fig. 4:**
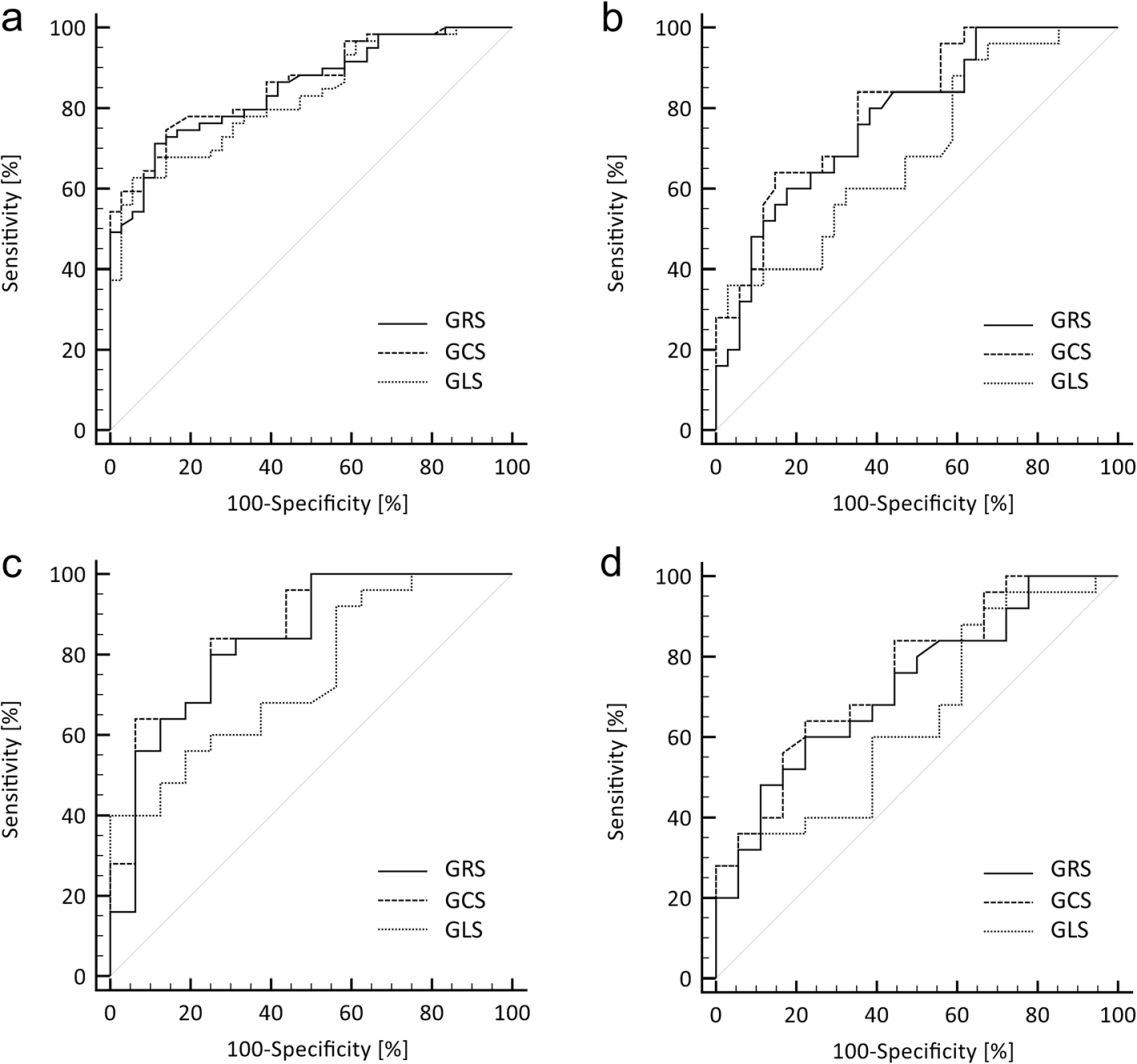
Strain: ROC Analysis. ROC curves for GRS, GCS and GLS comparing **a** All LVNC phenotype patients and healthy subjects ; **b** Subgroup A vs. B (LVNC phenotype patients with normal LV volumetrics and healthy subjects with normal LV volumetrics); **c** Subgroup A vs. C (LVNC phenotype patients with normal LV volumetrics and healthy subjects with normal LV volumetrics not meeting any CMR diagnostic criteria for LVNC); **d** Subgroup A vs. D (LVNC phenotype patients with normal LV volumetrics and healthy subjects with normal LV volumetrics meeting at least one of the CMR diagnostic criteria for LVNC). ROC = receiver operating characteristic; GRS = global radial strain; GCS = global circumferential strain; GLS = global longitudinal strain; LVNC = left ventricular non-compaction cardiomyopathy; LV = left ventricle; CMR = cardiovascular magnetic resonance

Reduced radial, circumferential and longitudinal strain in LVNC phenotype patients was observed compared to healthy subjects, with statistically significant differences at both global and slice specific levels (i.e. basal, mid and apical levels for radial and circumferential strain, and each LAX orientation for longitudinal strain). Comparisons between all LVNC phenotype patients and healthy subjects, as well as the subgroups, are illustrated with box plots in Fig. 3. There was no IQR overlap between patients and healthy controls in GRS (25th percentile of 33.4% for controls and 75th percentile of 32.7% for patients). IQR overlap was, however, observed between the groups for GCS and GLS. Results of the ROC analyses are detailed in Table 4. ROC curve analysis demonstrated an area under the curve (AUC) of 0.86 for GCS, 0.85 for GRS and 0.83 for GLS. ROC curves are illustrated in Fig. 4**.**

**Table 4:** ROC Analysis

	All LVNC phenotype patients and all healthy subjects	Subgroup A vs. B	Subgroup A vs. C	Subgroup A vs. D	Subgroup C vs. D
		LVNC phenotype patients (normal LV volumetrics) and healthy subjects (normal LV volumetrics)	LVNC phenotype patients (normal LV volumetrics) and healthy subjects (normal LV volumetrics, not meeting any LVNC criteria)	LVNC phenotype patients (normal LV volumetrics) and healthy subjects (normal LV volumetrics, meeting one or more LVNC criteria)	Healthy subjects (normal LV volumetrics, not meeting any LVNC criteria) and healthy subjects (normal LV volumetrics, meeting one or more LVNC criteria)
	AUC	AUC	AUC	AUC	AUC
GRS	0.85 (0.78, 0.92)	0.78 (0.66, 0.90)	0.84 (0.70, 0.97)	0.72 (0.57, 0.88)	0.65 (0.46, 0.84)
GCS	0.86 (0.79, 0.93)	0.80 (0.69, 0.91)	0.86 (0.74, 0.98)	0.76 (0.61, 0.90)	0.68 (0.49, 0.87)
GLS	0.83 (0.75, 0.91)	0.69 (0.56, 0.83)	0.75 (0.59, 0.90)	0.65 (0.48, 0.82)	0.65 (0.46, 0.84)

Data presented as AUC (95% CI)

*LVNC* left ventricular non-compaction cardiomyopathy, *LV* left ventricle, *ROC* receiver operating characteristic, *AUC* area under the curve, *GRS* global radial strain, *GCS* global circumferential strain, *GLS* global longitudinal strain, *CI* confidence interval

Comparing all healthy subjects and LVNC phenotype patients, GCS had independent and incremental diagnostic value beyond each of the CMR diagnostic criteria for LVNC: NC:C > 2.3 (OR 1.50, 95% CI: 1.04 to 2.18, *p* = 0.031, likelihood ration test χ^2^(df = 1) = 6.49, *p* = 0.011); NC mass > 20% (OR 1.69, 95% CI: 1.24 to 2.29, *p* = 0.001, likelihood ration test χ^2^(df = 1) = 18.15, *p* < 0.001); NC mass > 25% (OR 1.69, 95% CI: 1.04 to 2.73, *p* = 0.033, likelihood ration test χ^2^(df = 1) = 6.89, *p* = 0.009); NC mass > 15 g/m^2^ (OR 1.55, 95% CI: 1.17 to 2.07, *p* = 0.003, likelihood ratio test χ^2^(df = 1) = 13.39, *p* < 0.001).

##### Subgroup analyses

Statistically significant differences in GRS, GCS and GLS remained when LVNC phenotype patients were compared to healthy subjects with normal LV volumetrics (subgroup A vs. B) with AUCs of 0.80 for GCS, 0.78 for GRS and 0.69 for GLS, as well as when compared to healthy subjects not meeting any CMR diagnostic criteria for LVNC (subgroup A vs. C) with AUCs of 0.86 for GCS, 0.84 for GRS and 0.75 for GLS. Among subjects with normal LV volumetrics comparing LVNC phenotype patients with healthy subjects meeting at least one of the LVNC diagnostic criteria (subgroup A vs. D), statistically significant differences persisted for GCS (AUC 0.72) and GRS (AUC 0.69), but not GLS. However, only GCS remained significant in a bivariable logistic regression model after adjusting for EF (OR 1.59, 95% CI: 1.00 to 2.53, *p* = 0.049). In a nested logistic regression model with EF, model fit was significantly improved by the addition of GCS (χ^2^(df = 1) = 4.55, *p* = 0.033). No significant differences were demonstrated in global strain between healthy subjects with normal LV volumetrics meeting one or more LVNC criteria and those meeting none (subgroup C vs. D).

#### Torsion

Results for torsion are detailed in Table 3. No statistically significant differences were observed between all healthy subjects and LVNC phenotype patients or in the subgroup comparisons.

## Discussion

The results demonstrate abnormal myocardial strain in LVNC phenotype patients compared to healthy subjects, also present on comparison of subjects with normal LV volumetrics, although the study was unable to demonstrate differences independent of LV volumetrics known to affect myocardial strain. The differences were accentuated among subjects with normal LV volumetrics when LVNC phenotype patients were compared to healthy subjects not meeting any CMR diagnostic criteria for LVNC, but still persisted for GRS and GCS (GCS only when corrected for EF) when compared to healthy subjects with ‘physiologic’ hypertrabeculation (meeting one or more diagnostic criteria). Importantly, there were no significant differences between healthy subjects with and without physiologic hypertrabeculation. The AUCs for global strain values in differentiating LVNC phenotype patients from healthy subjects with ‘physiologic’ hypertrabeculation are reasonable considering the important limitations of morphology-based diagnostic criteria and GCS provides significant incremental value in addition to EF. GCS also adds significant incremental value when added to any of the four morphologic diagnostic criteria for LVNC among all healthy subjects and LVNC phenotype patients. No significant differences in LV torsion, however, were demonstrated by CMR FT analysis.

The significant proportion of healthy subjects meeting CMR diagnostic criteria for LVNC has previously been observed by larger studies. In the TASCFORCE (Tayside Screening for Cardiovascular Events) study 14.8% of subjects met at least one of the diagnostic criteria assessed, including an NC:C diameter ≥ 2.3 measured on end-diastolic LAX images (12.6%), ≥3 on end-diastolic SAO images (7.2%), ≥2 on end-systolic SAO images (4.4%), and NC > 20% of global LV mass (4.1%) [12]. The MESA (Multi Ethnic Study of Atherosclerosis) population observed an even higher proportion of asymptomatic subjects meeting criteria, with 43% of subjects demonstrating at least one segment with an NC:C diameter ratio > 2.3 on end-diastolic LAX images [13]. This study observed a particularly high proportion of healthy subjects exceeding previously validated diagnostic thresholds of non-compacted mass, likely related to the inherent limitations of contouring the borders of finely trabeculated non-compacted myocardium on 6–8 mm thick cine bSSFP SAO slices and inevitable inclusion of intertrabecular blood pool. Variability of measurement may partly account for differences in prevalence of healthy subjects meeting LVNC criteria between studies, particularly measurement of the NC:C diameter ratio where some studies have found poor inter-observer agreement depending on the technique used [12]; however, the remaining CMR diagnostic criteria based on measurement of the NC mass have more consistently shown a high degree of inter-observer reproducibility [10–12]. In any event, the high proportion of healthy subjects meeting morphology-based CMR diagnostic criteria emphasizes the important potential complementary diagnostic value of strain in differentiating LVNC from physiologic hypertrabeculation.

Although there are multiple studies describing the strain characteristics of LVNC assessed by STE [15–18], CMR FT has some potential advantages that may provide a more accurate and reproducible assessment. The comparatively unrestricted access to multi-planar views and excellent contrast between the myocardium and blood pool allows reproducible imaging planes and clear boundary points for the measurement of myocardial strain [19]. However, the relatively poor temporal resolution of routine bSSFP CMR precludes reliable assessment of more sophisticated time-resolved measures of strain, including strain rate and velocity.

Among the healthy subjects GRS values were slightly higher, and GCS and GLS slightly lower, compared to a meta-analysis of CMR FT derived strain values in normal subjects [23]. Although a high degree of inter-observer reproducibility in the CMR assessment of strain has been previously demonstrated [24], recognized variations in strain on images obtained at different field strengths and significant variability across post-processing software vendors [25] may account for these differences.

To the best of the authors’ knowledge, this study represents the largest group of LVNC phenotype patients assessed by CMR FT strain analysis. The findings of impaired myocardial strain among LVNC phenotype patients are consistent with previous smaller studies [26, 27]. A study by Cai J et al. investigated the relationship between LV hypertrabeculation measured by fractal analysis and myocardial deformation by CMR FT among 180 healthy subjects and 10 LVNC patients, demonstrating an independent association between the degree of hypertrabeculation and reduced circumferential strain [26]. A retrospective study by Nucifora et al demonstrated impairment of radial, circumferential and longitudinal strain assessed by CMR FT in separate groups of children/adolescents and young adults with a total of 32 isolated LVNC patients compared to age-matched controls [27]. Impairment of myocardial deformation in the children/adolescent group with preserved LVEF but comparable degrees of non-compaction to young adult patients with reduced LVEF was observed, suggesting that abnormal strain may predict the development of clinically overt cardiomyopathy in LVNC.

The absence of a significant difference in LV twist and torsion is inconsistent with previous STE studies demonstrating significantly decreased twist in LVNC phenotype patients compared to healthy subjects [17, 18]. A disadvantage of CMR FT compared to STE that may partly account for these differences is the fairly uniform water content and thus homogenous signal within myocardium on bSSFP sequences. This may preclude accurate tracking of features within the myocardium required to measure rotational circumferential displacement and therefore LV twist, whereas other strain parameters dependent on tracking well-defined endo- and epicardial borders appear to be more robust [19]. Nevertheless, significantly greater degrees of LV torsion have been demonstrated amongst healthy subjects in previous STE [28] and CMR FT studies [29], with reported normal torsion values of 2.7°/cm (standard deviation ±1.5) at CMR FT compared to 0.94°/cm (IQR: 0.74, 1.54) observed in our study. These differences may again be related to differences in tissue tracking techniques employed by different post-processing software vendors.

### Limitations

LVNC is a rare cardiomyopathy with a variety of clinical manifestations and growing number of genetic associations described in recent years that demonstrate phenotypic overlap with other cardiomyopathies [30]. Due to the limited number of LVNC phenotype patients, it was beyond the scope of this study to investigate potential associations between clinical manifestations and strain. Furthermore, it was not possible to limit the study to genetically confirmed cases as a minority of patients will have undergone testing for multiple associated genes. The retrospective nature of the study may have introduced an element of selection bias, as LVNC patients identified from clinic databases and CMR reports are more likely to have more severe disease. A further limitation is the use of a single post-processing software vendor for CMR FT strain analysis, as previous studies have demonstrated significant inter-vendor variability [25]; cut-points have therefore not been provided due to limited clinical applicability.

## Conclusions

LVNC phenotype patients demonstrate impaired radial, circumferential and longitudinal strain by CMR FT, persistent on comparison of subjects with normal LV volumetrics meeting at least one diagnostic criteria for LVNC. The high proportion of healthy subjects meeting morphology-based CMR diagnostic criteria emphasizes the important potential complementary diagnostic value of strain in differentiating LVNC from physiologic hypertrabeculation.

## Data Availability

The datasets used in the study are available from the corresponding author on reasonable request.

## Abbreviations

AUC: Area under the curve
BSA: Body surface area
bSSFP: Balanced steady-state free precession
C: Compacted myocardium
CMR: Cardiovascular magnetic resonance
EDV: End-diastolic volume
EDVI: End-diastolic volume indexed to body surface area
EF: Ejection fraction
ESV: End-systolic volume
ESVI: End-systolic volume indexed to body surface area
FT: Feature tracking
GCS: Global circumferential strain
GLS: Global longitudinal strain
GRS: Global radial strain
IQR: Interquartile range
LAX: Long-axis
LV: Left ventricle/left ventricular
LVNC: Left ventricular non-compaction cardiomyopathy
MASSi: Myocardial mass indexed to body surface area
NC: Non-compacted myocardium
ROC: Receiver operating characteristic
SAO: Short-axis oblique
STE: Speckle-tracking echocardiography
SV: Stroke volume
SVI: Stroke volume indexed to body surface area
